# Correction: Surfactant-chaperoned donor–acceptor–donor NIR-II dye strategy efficiently circumvents intermolecular aggregation to afford enhanced bioimaging contrast

**DOI:** 10.1039/d6sc90215d

**Published:** 2026-11-09

**Authors:** Tianyang Han, Yajun Wang, Jiajun Xu, Ningning Zhu, Lang Bai, Xiangping Liu, Bin Sun, Chenlong Yu, Qinglun Meng, Jiaqi Wang, Qi Su, Qing Cai, Kenneth S. Hettie, Yuewei Zhang, Shoujun Zhu, Bai Yang

**Affiliations:** a State Key Laboratory of Supramolecular Structure and Materials, College of Chemistry, Jilin University Changchun 130012 P. R. China sjzhu@jlu.edu.cn; b Joint Laboratory of Opto-Functional Theranostics in Medicine and Chemistry, First Hospital of Jilin University Changchun 130021 P. R. China; c Hospital of Stomatology, Jilin University Changchun 130021 P. R. China; d Molecular Imaging Program at Stanford (MIPS), Department of Radiology, Stanford University School of Medicine Stanford California 94305 USA; e School of Chemistry and Pharmaceutical Engineering, Jilin Institute of Chemical Technology Jilin 132022 P. R. China

## Abstract

Correction for ‘Surfactant-chaperoned donor–acceptor–donor NIR-II dye strategy efficiently circumvents intermolecular aggregation to afford enhanced bioimaging contrast’ by Tianyang Han *et al.*, *Chem. Sci.*, 2022, **13**, 13201–13211, https://doi.org/10.1039/d2sc05651h.

It has come to the author's attention that an error requiring correction exists in Fig. 3g. The liver H&E data in Fig. 3g were incorrectly assembled. Although this does not affect the validity of the article's conclusions, to avoid potential misunderstandings, we have provided a corrected Fig. 3g in this correction. In fact, we performed triplicate experiments (*n* = 3) for Control, IR-FH4P, and IR-FH4P/Triton, and all results were negative. We sincerely apologize for this error. The correct figure is displayed below.

**Fig. 3:**
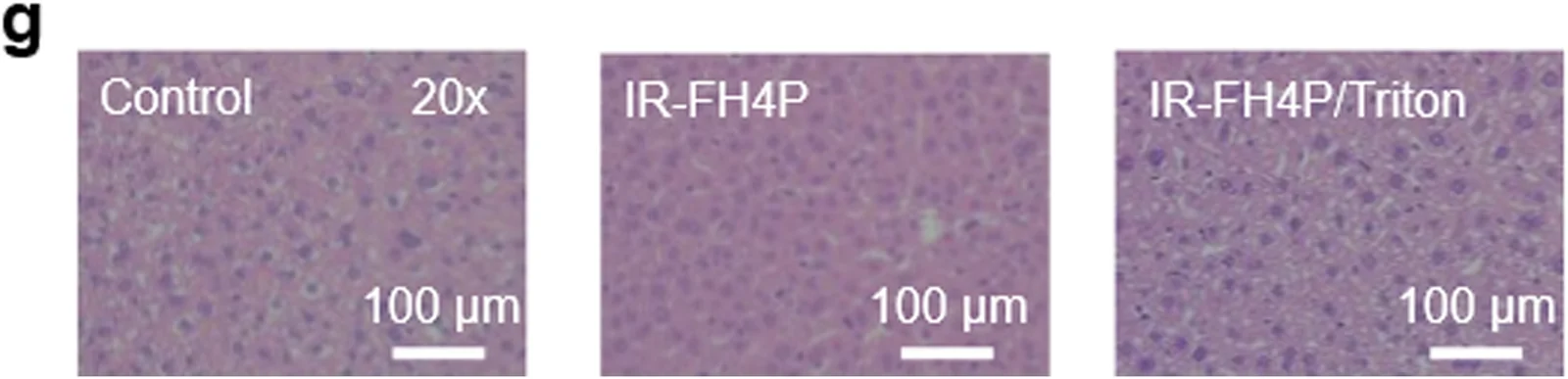
(g) Histological evaluation of the liver after intravenous administration of 24 hours of PBS, IR-FH4P, and IR-FH4P/Triton.

The Royal Society of Chemistry apologises for these errors and any consequent inconvenience to authors and readers.

